# Supplementary figures and images for: A major trade-off between growth and defense in Arabidopsis thaliana can vanish in field conditions
Source: PLoS Biol. 2025 Jul 14;23(7):e3003237. doi: 10.1371/journal.pbio.3003237 (PMC12273917; doi:10.1371/journal.pbio.3003237)

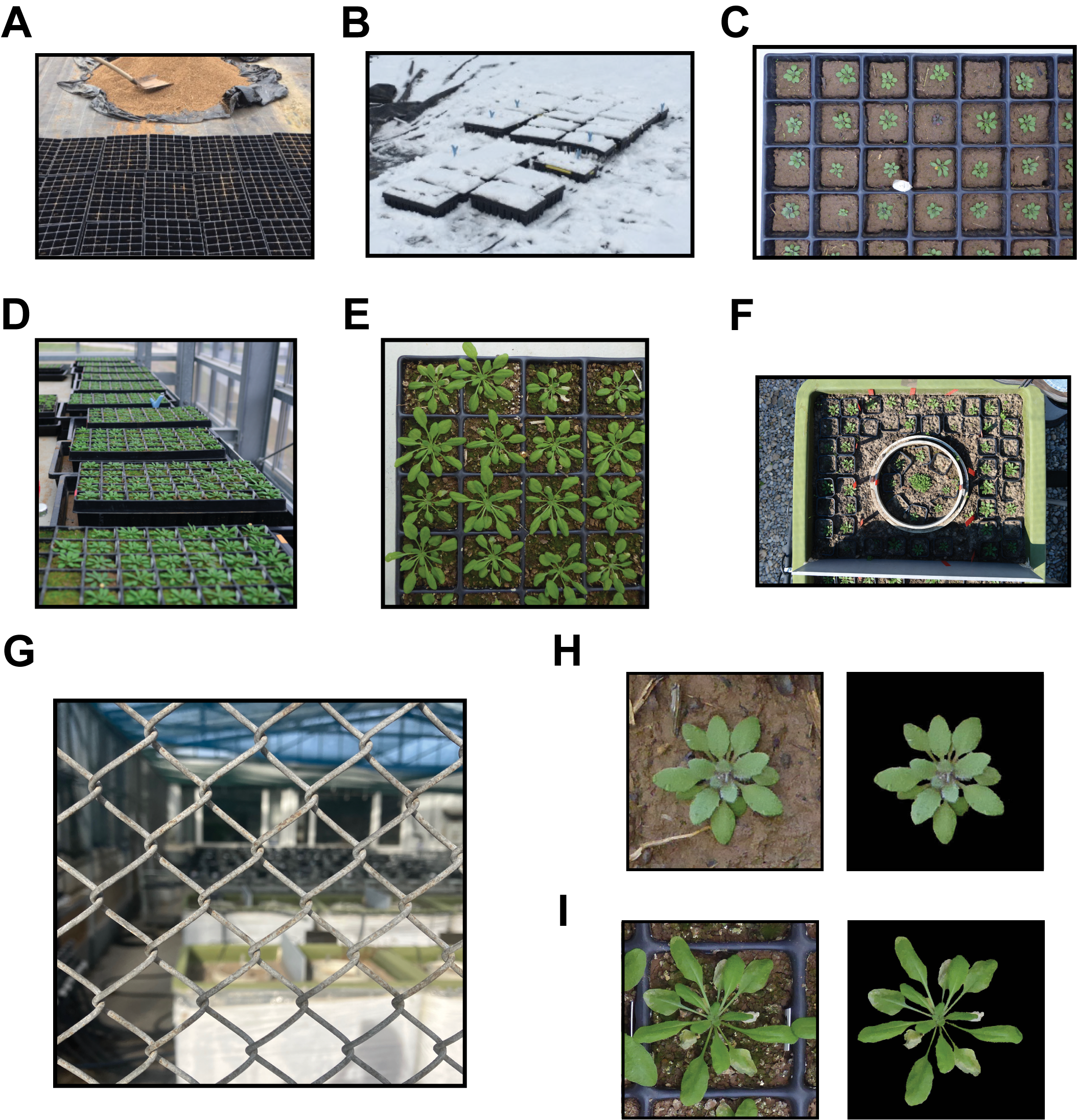

Supplement: S1 Fig — (A) Sieved and homogenized field soil ready for distribution into pots for experiments in Tübingen. (B) Field-grown plants in Tübingen under snowfall in January. (C) Field-grown plants in Tübingen from 2016 to 2017 season on the day of harvest. (D) Greenhouse-grown plants in Tübingen from 2016 to 2017 season. (E) Greenhouse-grown plants in Tübingen from 2016 to 2017 season on the day of harvest. (F) Field-grown plants in Zurich from 2018 to 2019. (G) Closeup of a chain-link fence as an open side partition at the open-air room at the Zurich field site. The ceiling was open except for a net. Wind, rain, and insects could freely pass through. (H) Images of field plants from Tübingen on day or harvest before (left) and after (right) background removal. (I) Images of greenhouse plants from Tübingen on day or harvest before (left) and after (right) background removal. Note that overlap of leaves from plants in adjacent pots occasionally required manual estimation of leaf borders. (TIF) [file pbio.3003237.s001.tif]

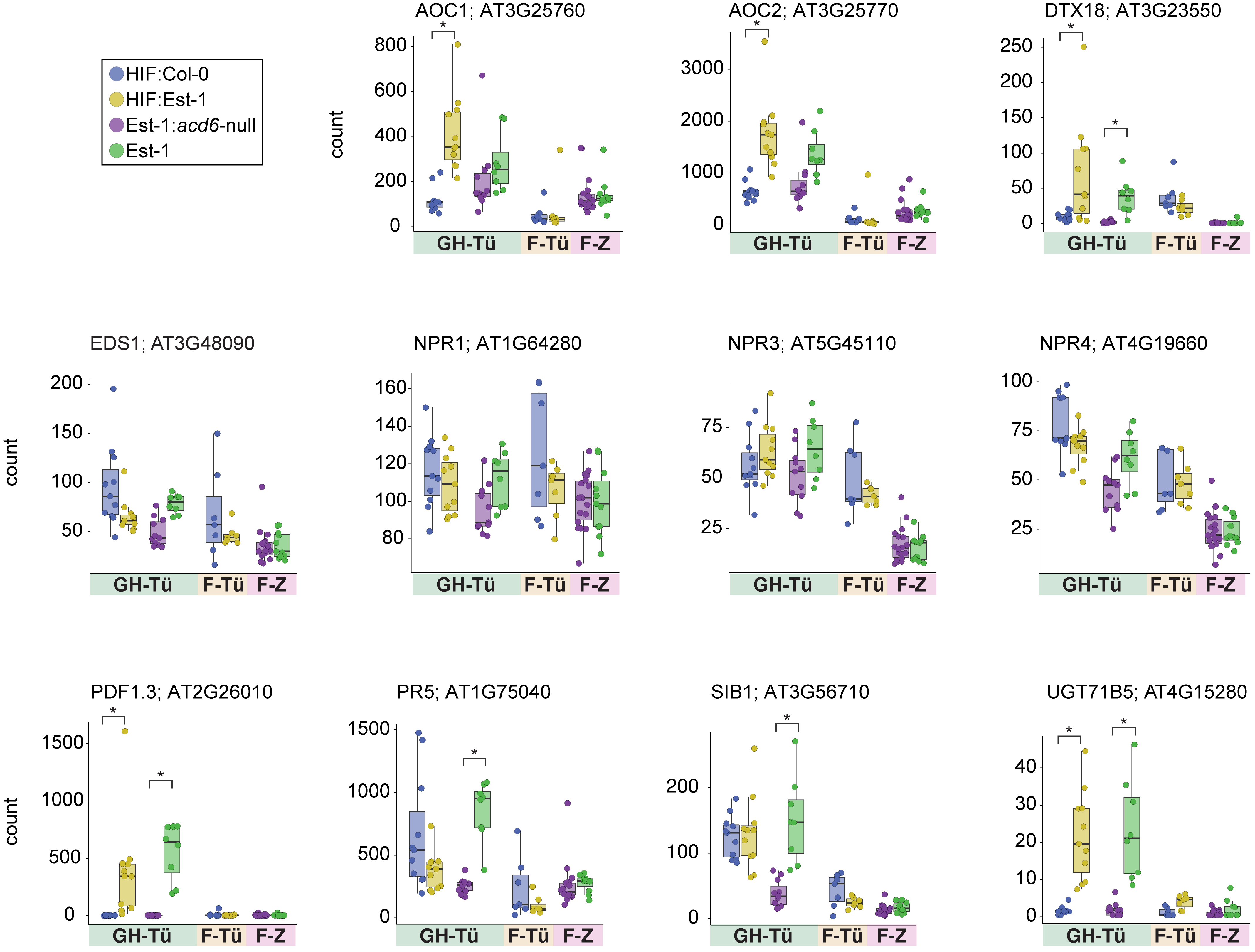

Supplement: S2 Fig — Normalized RNA-seq read counts (y-axes) for different genes with relevance to immune system activity. Colors of boxplots indicate plant genotype as described at the top left. The colored annotations along the x-axis denote the environment (GH-Tü = Greenhouse Tübingen, F-Tü = Field Tübingen, F-Z = Field Zurich). * signifies P < 0.01 in an FDR-corrected Wald test. The data underlying this figure can be found in https://doi.org/10.5281/zenodo.15527338. (TIF) [file pbio.3003237.s002.tif]

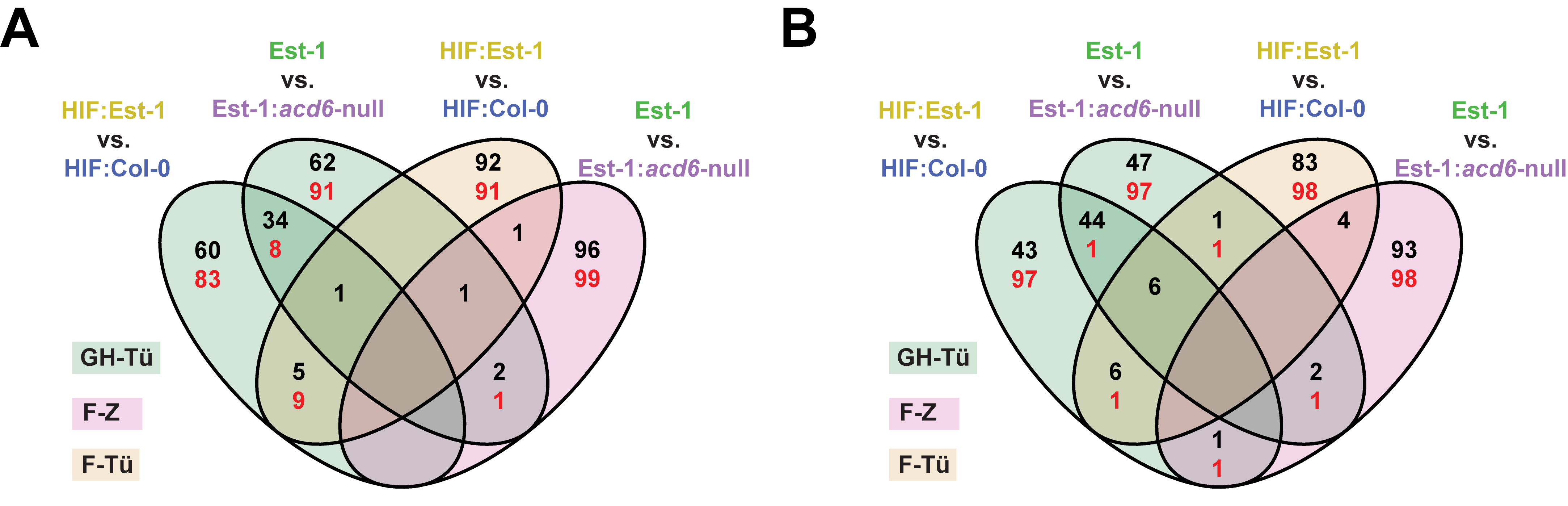

Supplement: S3 Fig — (A) Overlaps between upregulated (black numbers) and downregulated (red numbers) genes in ACD6 allelic contrasts, either in the greenhouse (top) or field (bottom), for the 100 genes with the lowest P values from each contrast (regardless of any significance threshold). (B) Similar to A, but for the 100 genes from each contrast with the largest fold change (regardless of any significance threshold). The data underlying this figure can be found in https://doi.org/10.5281/zenodo.15527338. (TIF) [file pbio.3003237.s003.tif]

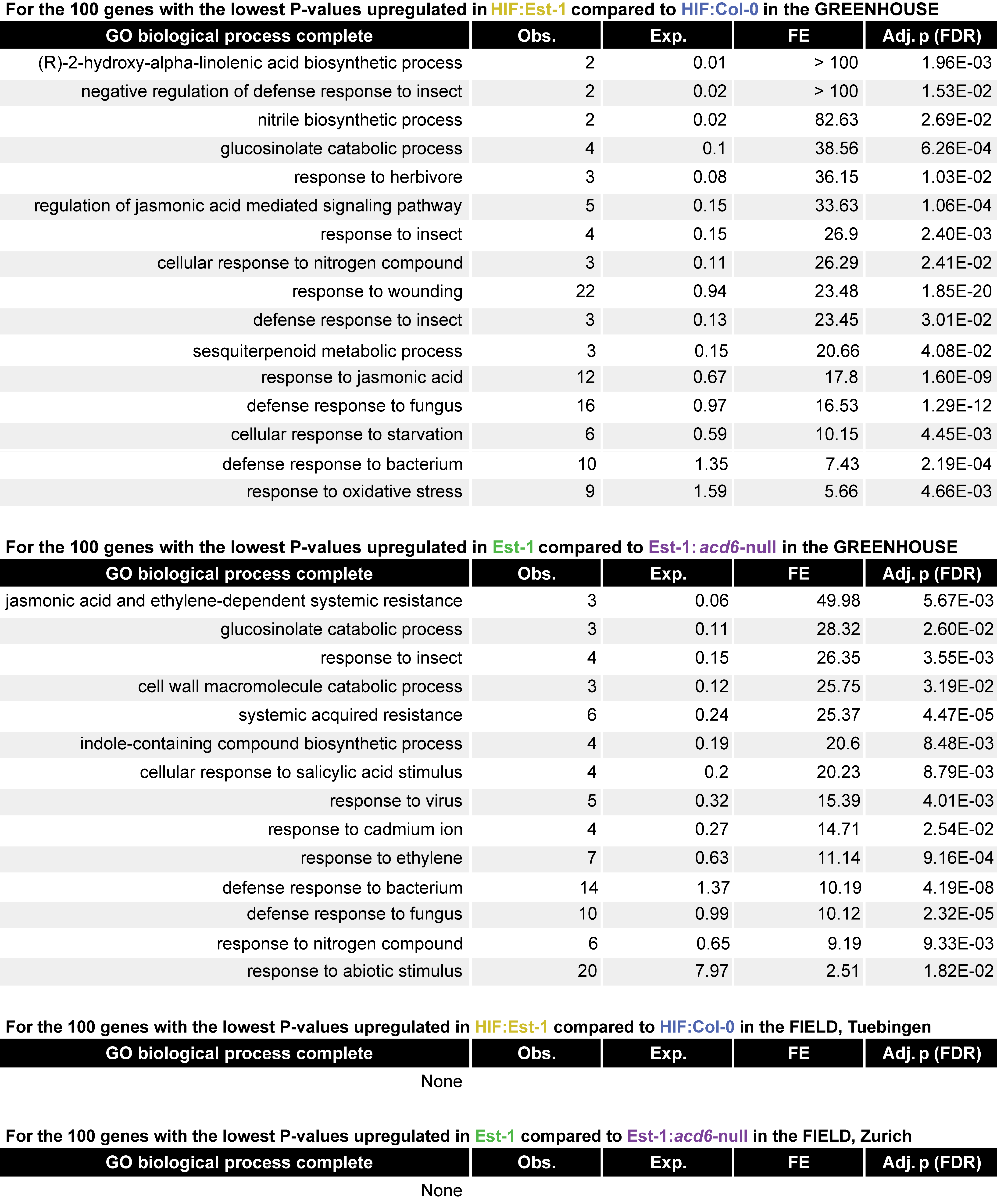

Supplement: S4 Fig — The top 100 upregulated genes are defined as those with the lowest P-values, regardless of a P-value threshold. In both greenhouse comparisons, multiple immune system processes are significantly enriched among these 100 genes. In both field comparisons, no biological process is significantly enriched among these 100 genes. Obs., bserved count; Exp., Expected count; FE, Fold enrichment; Adj. P is the FDR-corrected P-value. (TIF) [file pbio.3003237.s004.tif]

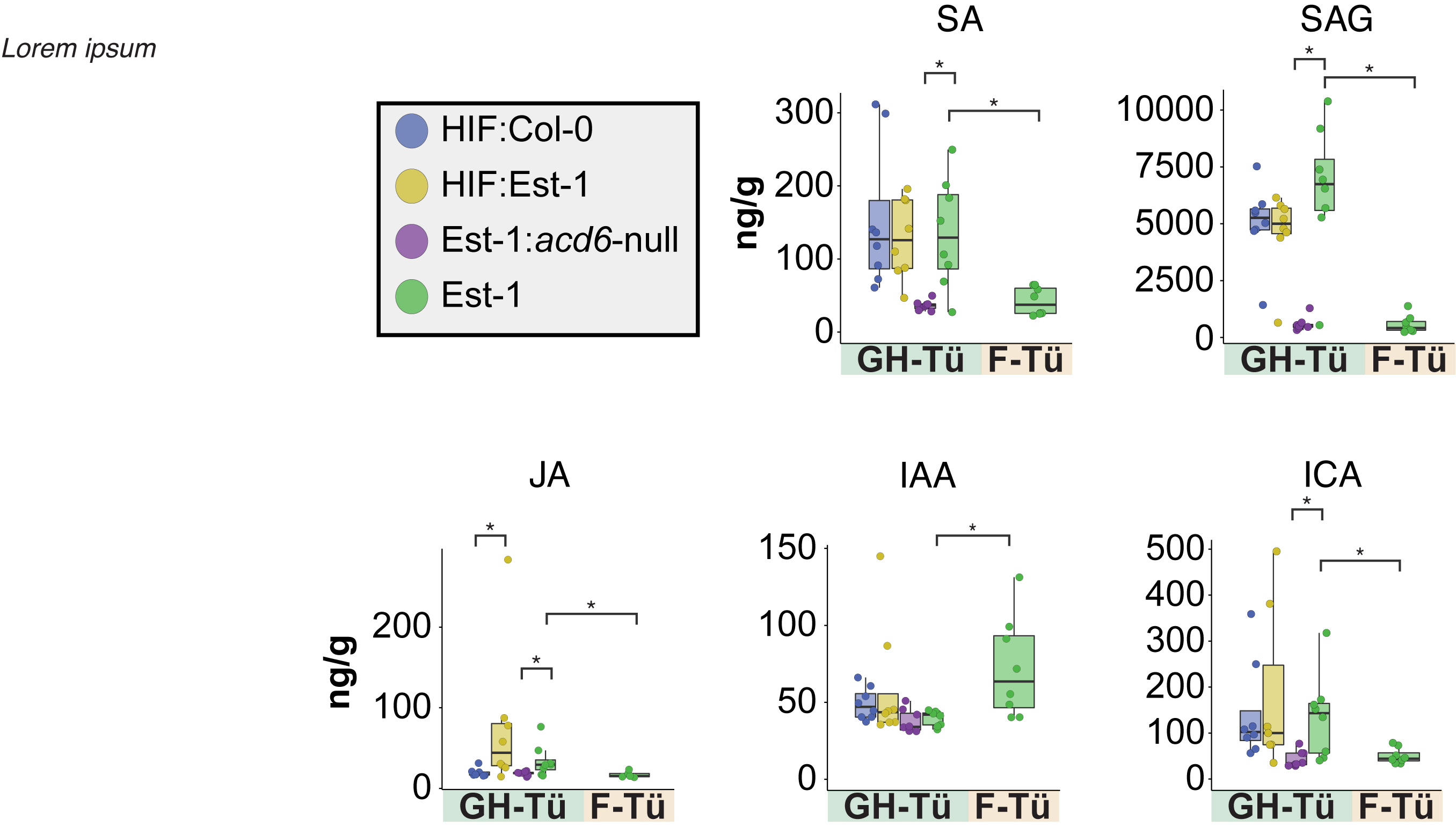

Supplement: S5 Fig — From rosettes grown in Tübingen, salicylic acid (SA), its inactivate storage form SA O-β-glucoside (SAG), jasmonic acid (JA), indole-3-acetic acid (IAA), and indole-3-carboxylic acid (ICA) were measured from snap-frozen tissue via LC-MS. Letters “a” and “b” above the boxplots represent groups that are statistically different in a FDR-corrected Mann–Whitney U-test (P < 0.05) across three comparisons: HIF:Col-0 vs. HIF:Est-1 in the greenhouse, Est-1:acd6-null vs. Est-1 in the greenhouse, and Est-1 in the greenhouse vs. Est-1 in the field. The data underlying this figure can be found in https://doi.org/10.5281/zenodo.15527338. (TIF) [file pbio.3003237.s005.tif]

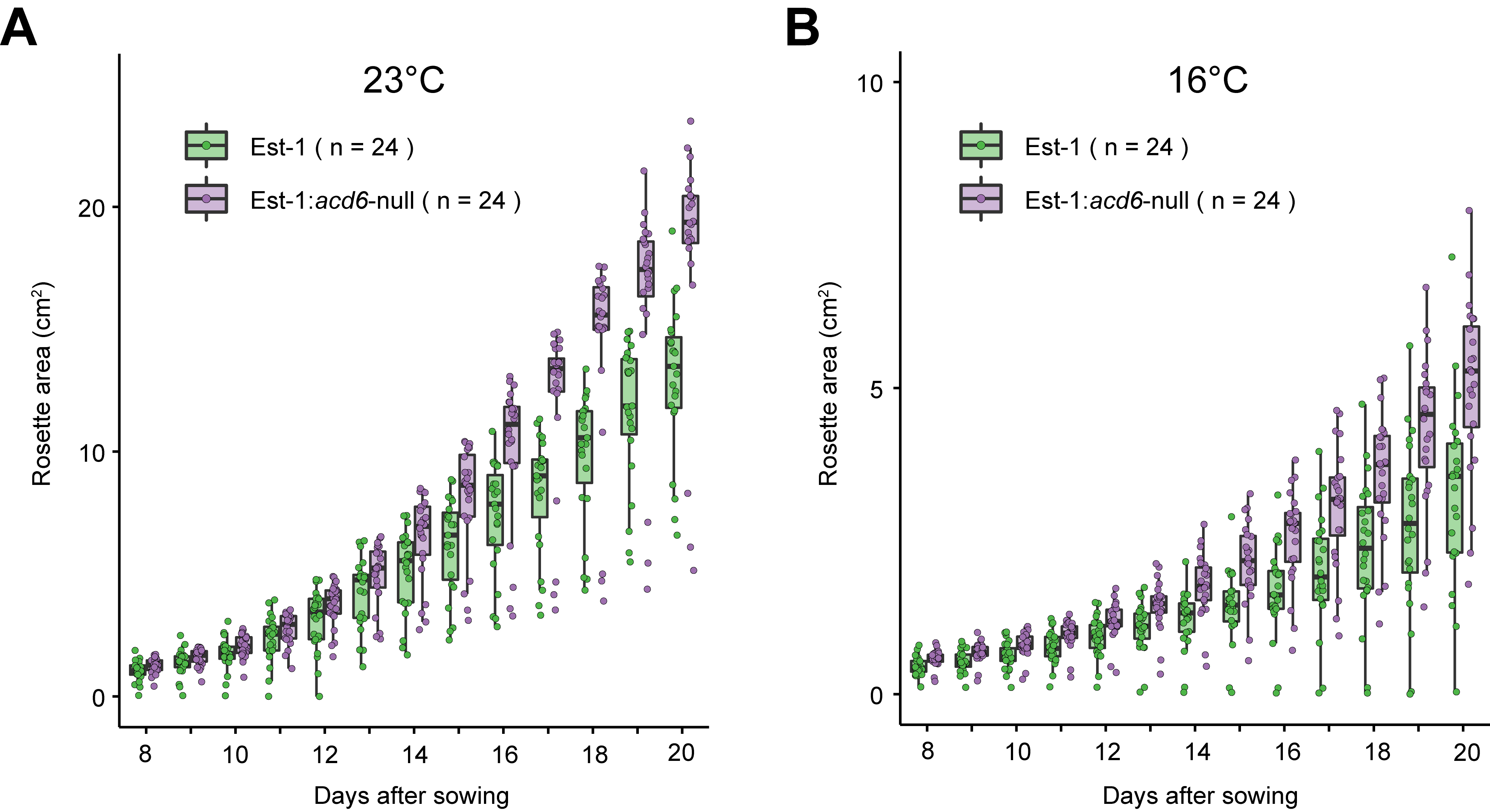

Supplement: S6 Fig — Twenty-four Est-1 and 24 Est-1:ACD6-null seeds were sown in potting soil, grown in either 16 or 23°C long (16 h) days, and green pixels were monitored daily from overhead photographs starting 1 week after sowing. In both temperature regimes. The data underlying this figure can be found in https://doi.org/10.5281/zenodo.15527338. (TIF) [file pbio.3003237.s006.tif]

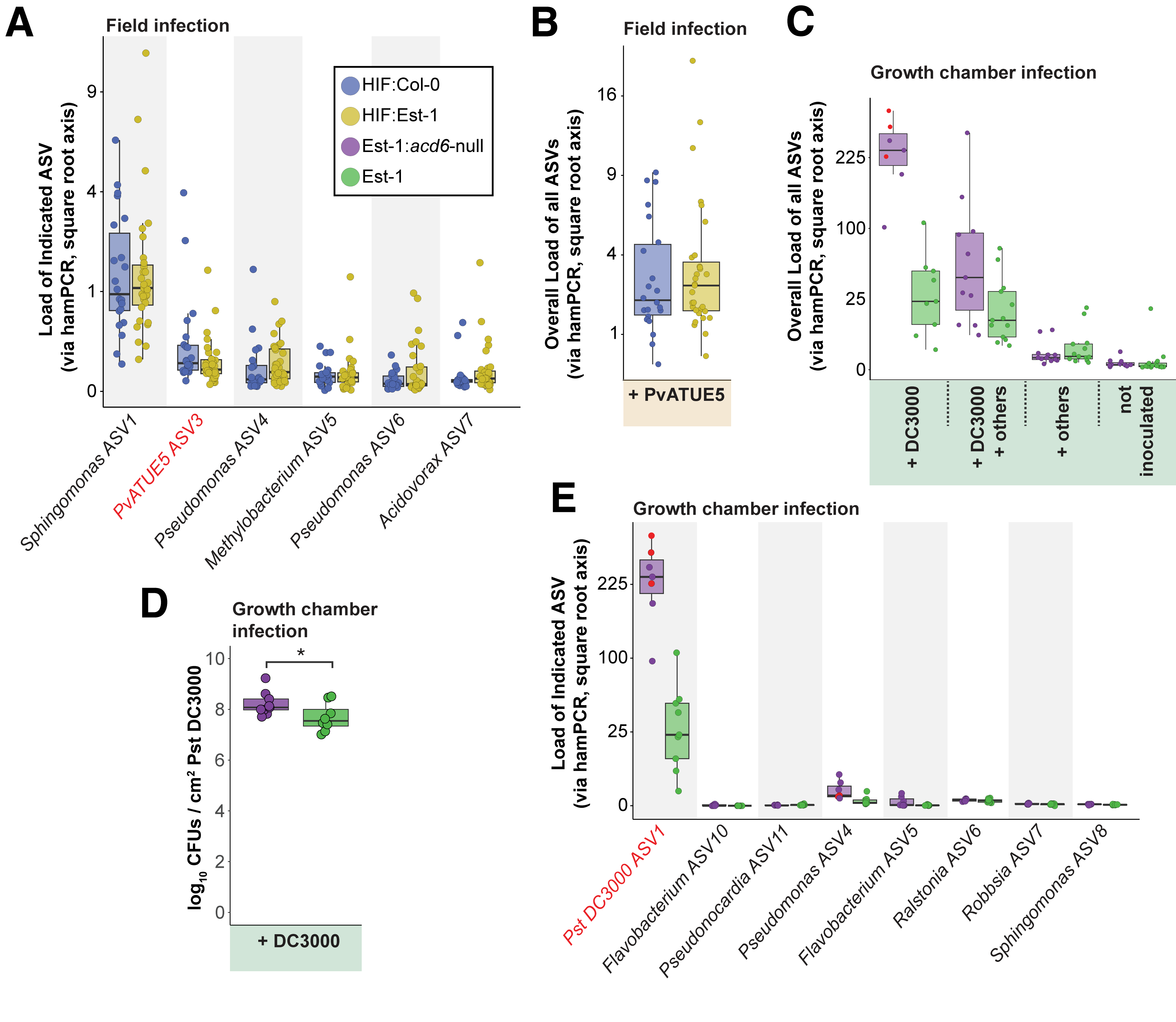

Supplement: S7 Fig — (A) Bacterial loads of the most abundant ASVs in field-grown HIF plants challenged with a cocktail of PvATUE5 strains in the field for 1 week, with color conventions for the plant genotypes as shown in the legend. The ASV corresponding to the cocktail of PvATUE5 is labeled in red. Boxes enclose the interquartile range (IQR) with whiskers extending to up to 1.5 times the IQR. (B) Overall bacterial load considering all ASVs for the plants in (A). (C) Overall bacterial load for all ASVs for growth chamber-grown Est-1 and Est-1:acd6-null plants challenged with Pst DC3000 and/or other phyllosphere bacteria for 4 days, with color code as in (A). Red points indicate samples with especially high bacterial load for which hamPCR could not provide accurate quantification due to a small number of reads from the plant’s GIGANTEA gene. d, Colony forming units (CFUs) of Pst DC3000 per cm2 of leaf tissue for Pst DC3000-only infected plants shown in (C). * indicates P < 0.05 in a Mann–Whitney U-test. (E) Bacterial loads of the most abundant ASVs in growth chamber-grown plants infected with Pst DC3000 only as shown in (C) and (D). Red points indicate samples with especially high bacterial load for which hamPCR could not provide accurate quantification due to a small number of reads from the plant’s GIGANTEA gene. Sample size without considering the red points is too small and statistics are, therefore, omitted. The ASV corresponding to Pst DC3000 is labeled in red. The data underlying this figure can be found in https://doi.org/10.5281/zenodo.15527338. (TIF) [file pbio.3003237.s007.tif]

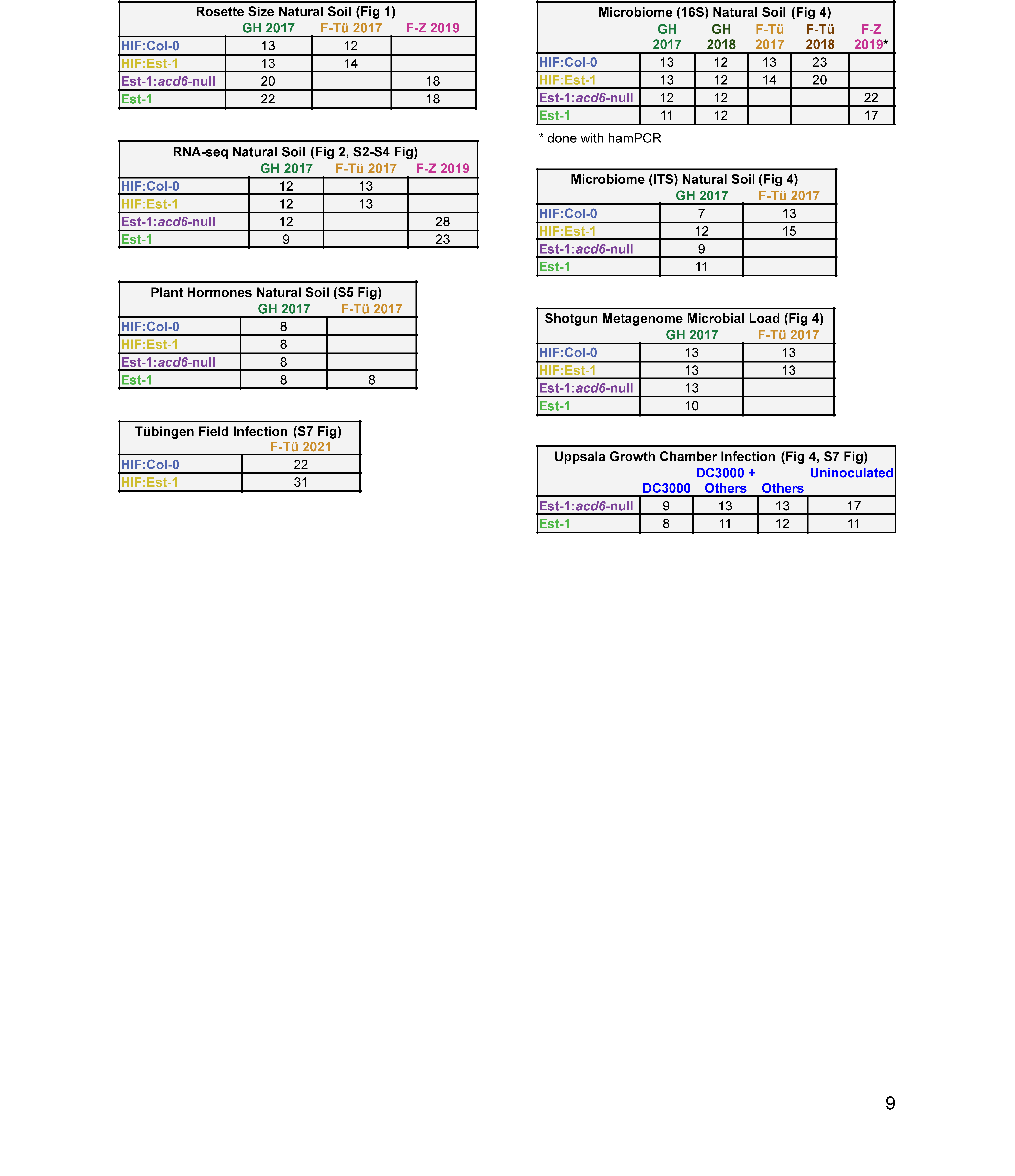

Supplement: S8 Fig — (TIF) [file pbio.3003237.s008.tif]
